# Supplementary material for: Breastfeeding Practices, Infant Formula Use, Complementary Feeding and Childhood Malnutrition: An Updated Overview of the Eastern Mediterranean Landscape
Source: Nutrients. 2022 Oct 9;14(19):4201. doi: 10.3390/nu14194201 (PMC9572091; doi:10.3390/nu14194201)
Supplement: Supplementary file 1 [file nutrients-14-04201-s001.zip › nutrients-1874980-supplementary.pdf]

Table S1: National, regional, and global prevalence of feeding patterns and malnutrition among under 5 years children.

Countries	Prevalence of infant feeding						Prevalence of malnutrition					References
	EvBF (%)	EBF (%)	BOT (%)	CBF (%)	MixMF (%)	ISSSF (%)	Stunting (%)	Under-weight (%)	Wasting (%)	Overweight (%)	Obesity (%)	
**Afghanistan (2018)**	NA	57.5	NA	NA	NA	NA	38.2	NA	5.1	4.1	NA	[36]
**Afghanistan (2015)**	NA	NA	NA	NA	NA	56	NA	NA	NA	NA	NA	[38]
**Afghanistan (2014)**	NA	NA	NA	NA	NA	NA	NA	NA	28.3	NA	NA	[98]
**Afghanistan (UNICEF/WHO/ World Bank database, 2000-2020)**	NA	In 2018: 57.5	NA	In 2015: 73.8	NA	In 2015: 61	In 2020: 35.1	In 2018: 19.1	In 2018: 5.1	In 2020: 3.9	NA	[35,37,92,99,104, 106]
**Bahrain (2009)**	NA	NA	NA	NA	NA	NA	NA	NA	NA	9.9	6.5	[107]
**Bahrain (UNICEF/WHO/ World Bank database, 2000-2020)**	NA	NA	NA	NA	NA	NA	In 2020: 5.1	In 1995: 7.6	In 1995: 6.6	In 2020: 6.4	NA	[35,37,92,99,104, 106]
**Djibouti (2012)**	NA	12.4	NA	NA	NA	NA	33.5	NA	21.5	8.1	NA	[39]

Countries	Prevalence of infant feeding						Prevalence of malnutrition					References
	EvBF (%)	EBF (%)	BOT (%)	CBF (%)	MixMF (%)	ISSSF (%)	Stunting (%)	Under-weight (%)	Wasting (%)	Overweight (%)	Obesity (%)	
**Djibouti (UNICEF/WHO/ World Bank database, 2000-2020)**	NA	In 2017: 12.4	NA	NA	NA	NA	In 2020: 34	In 2013: 26	In 2012: 21.5	In 2020: 7.2	NA	[35,37,92,99,104, 106]
**Egypt (2017-2018)**	97.3	18.5	At ≤1m: 72.5<br>1-2m: 12.1<br>At ≥2m: 15.4	NA	NA	NA	NA	NA	NA	NA	NA	[26]
**Egypt (2018)**	NA	NA	31	NA	69	NA	NA	NA	NA	NA	NA	[40]
**Egypt (2017)**	NA	14.1	NA	NA	NA	NA	NA	NA	NA	NA	NA	[43]
**Egypt (2014-2015)**	NA	49.7	37.6	NA	12.7	NA	NA	NA	NA	NA	NA	[41]
**Egypt (2014)**	NA	39.5	NA	Up to 1y: 80<br>Up to 2y: 20.4	NA	75.2	22.3	NA	9.5	15.7	NA	[42]
**Egypt (2014)**	NA	NA	NA	NA	NA	75.2	21	5.7	9.2	4.4	NA	[45]
**Egypt (2014)**	NA	NA	NA	NA	NA	NA	18.5	23.2	19.3	NA	NA	[112]
**Egypt (2013)**	NA	32.2	NA	NA	NA	NA	NA	NA	NA	NA	NA	[44]

Countries	Prevalence of infant feeding						Prevalence of malnutrition					References
	EvBF (%)	EBF (%)	BOT (%)	CBF (%)	MixMF (%)	ISSSF (%)	Stunting (%)	Under-weight (%)	Wasting (%)	Overweight (%)	Obesity (%)	
**Egypt (2012)**	100	29.9	NA	NA	NA	63.6	NA	NA	NA	NA	NA	[28]
**Egypt (2010)**	95.8	9.7	NA	NA	86.1	NA	NA	NA	NA	NA	NA	[27]
**Egypt (2008)**	NA	NA	NA	NA	NA	NA	In 2008: 28.9	In 2008: 6	In 2008:7.2	NA	NA	[100]
**Egypt (UNICEF/WHO/ World Bank database, 2000-2020)**	NA	In 2014: 39.5	NA	In 2014: 50.5	NA	In 2014: 75.2	In 2020: 22.3	In 2014: 7	In 2014: 9.5	In 2020: 17.8	NA	[35,37,92,99,104, 106]
**Iran (2018)**	NA	NA	NA	NA	NA	NA	8.45	7.63	8.04	1.23	NA	[102]
**Iran (2014-2015)**	98.6	At discharge: 74.3<br>At 6m: 28	NA	NA	At discharge: 23<br>At 6m: 87	NA	NA	NA	NA	NA	NA	[46]
**Iran (2011)**	NA	NA	NA	NA	NA	NA	7.3	2.3	1.4	5.1	1.3	[108]
**Iran (2010)**	NA	53.1	NA	Up to 1y: 84.2<br>Up to 2y: 51	NA	75.9	6.8	NA	4	NA	NA	[47]

Countries	Prevalence of infant feeding						Prevalence of malnutrition					References
	EvBF (%)	EBF (%)	BOT (%)	CBF (%)	MixMF (%)	ISSSF (%)	Stunting (%)	Under-weight (%)	Wasting (%)	Overweight (%)	Obesity (%)	
**Iran (2010)**	NA	NA	NA	NA	NA	NA	10.13	5.7	3.2	NA	NA	[113]
**Iran (2010)**	NA	53.13	31.15	NA	NA	75.92	NA	NA	NA	NA	NA	[48]
**Iran (2009-2010)**	NA	NA	NA	NA	NA	NA	5.8	4.8	10.3	NA	NA	[103]
**Iran (2007-2008)**	NA	61	NA	NA	NA	NA	NA	NA	NA	NA	NA	[49]
**Iran (2007)**	NA	NA	NA	NA	NA	NA	12.7	11.7	5.9	NA	NA	[114]
**Iran (2005-2006)**	NA	At 4m: 56.8<br>At 6m: 27.7	NA	Up to 1y:90<br>Up to 2y:57	NA	NA	NA	NA	NA	NA	NA	[50]
**Iran (2004)**	NA	NA	NA	NA	NA	NA	12.5	7.5	4.4	NA	NA	[115]
**Iran (UNICEF/WHO/ World Bank database, 2000-2020)**	NA	In 2010: 53.1	NA	NA	NA	In 2010: 75.9	In 2020: 6.3	In 2017: 4.3	In 2010: 4	In 2020: 9.4	NA	[35,37,92,99,104, 106]
**Iraq (2018)**	NA	25.8	NA	Up to 1y: 44.8<br>Up to 2y: 26.7	NA	84.8	12.6	NA	3	6.1	NA	[52]
**Iraq (2017-2018)**	NA	26.5	NA	NA	NA	NA	NA	NA	NA	NA	NA	[55]

Countries	Prevalence of infant feeding						Prevalence of malnutrition					References
	EvBF (%)	EBF (%)	BOT (%)	CBF (%)	MixMF (%)	ISSSF (%)	Stunting (%)	Under-weight (%)	Wasting (%)	Overweight (%)	Obesity (%)	
**Iraq (2016)**	NA	NA	NA	NA	NA	NA	20.6	7.6	6.6	NA	NA	[117]
**Iraq (2015)**	88.5	27	NA	NA	NA	NA	NA	NA	NA	NA	NA	[54]
**Iraq (2012-2013)**	NA	31	34	35	NA	78	NA	NA	NA	NA	NA	[53]
**Iraq (2009)**	NA	NA	NA	NA	NA	NA	NA	18.2	NA	NA	NA	[116]
**Iraq (2006-2012)**	NA	In 2006: 35<br>In 2012: 39.7	In 2006: 37<br>In 2012: 27	NA	In 2006: 29.8<br>In 2012: 22.5	NA	In 2006: 51.2<br>In 2012: 30.9	NA	NA	In 2006: 20<br>In 2012: 7.2		[51]
**Iraq (UNICEF/WHO/ World Bank database, 2000-2020)**	NA	In 2018: 25.8	NA	In 2018: 35.3	NA	In 2018: 84.8	In 2020: 11.6	In 2018: 3.9	In 2018: 3	In 2020: 9	NA	[35,37,92,99,104, 106]
**Jordan (2012-2017)**	NA	In 2017: 25.4	NA	In 2017:<br>Up to 1y: 36.2<br>Up to 2y: 14.9	NA	In 2017: 83.4	In 2012: 7.8	NA	In 2012: 2.4	In 2012: 4.7	NA	[57]
**Jordan (2017)**	71.2	20.9	NA	NA	NA	NA	NA	NA	NA	NA	NA	[58]
**Jordan (2017)**	87	At birth: 51<br>At 1m: 47<br>At 6m: 33	NA	>6m: 50	At birth:36<br>At 1m: 38<br>At 6m: 43	NA	NA	NA	NA	NA	NA	[56]

Countries	Prevalence of infant feeding						Prevalence of malnutrition					References
	EvBF (%)	EBF (%)	BOT (%)	CBF (%)	MixMF (%)	ISSSF (%)	Stunting (%)	Under-weight (%)	Wasting (%)	Overweight (%)	Obesity (%)	
**Jordan (2014)**	95	1	NA	NA	NA	NA	NA	NA	NA	NA	NA	[59]
**Jordan (UNICEF/WHO/ World Bank database, 2000-2020)**	NA	In 2017: 25.4	NA	In 2017: 26.1	NA	In 2017: 83.4	In 2020: 7.3	In 2019: 2.7	In 2012: 2.4	In 2020: 7.1	NA	[35,37,92,99,104, 106]
**Kuwait (2016-2019)**	NA	NA	NA	NA	NA	NA	NA	NA	NA	10.9	3.7	[118]
**Kuwait (2017)**	NA	NA	NA	NA	NA	NA	6.4	NA	2.5	5.5	NA	[111]
**Kuwait (2015)**	98.1	At discharge: 36.5	At discharge: 26.5	NA	At discharge: 37	NA	NA	NA	NA	NA	NA	[32]
**Kuwait (2012-2013)**	90.4	At 6m: 26.5	NA	NA	NA	NA	NA	NA	NA	NA	NA	[33]
**Kuwait (2007-2008)**	NA	At 4m : 31<br>At 8m : 26	At discharge : 8.4	At 12m : 22<br>At 26m : 2	At dicharge : 59.4	NA	NA	NA	NA	NA	NA	[60]
**Kuwait (2007-2008)**	92.5	At discharge : 10.5	At discharge : 15.3	NA	At dicharge : 55	NA	NA	NA	NA	NA	NA	[34]
**Kuwait (UNICEF/WHO/ World Bank**	NA	NA	NA	NA	NA	NA	In 2020: 6	In 2014: 3	In 2017: 2.5	In 2020: 7.1	NA	[35,37,92,99,104, 106]

Countries	Prevalence of infant feeding						Prevalence of malnutrition					References
	EvBF (%)	EBF (%)	BOT (%)	CBF (%)	MixMF (%)	ISSSF (%)	Stunting (%)	Under-weight (%)	Wasting (%)	Overweight (%)	Obesity (%)	
**database, 2000-2020)**												
**Lebanon (2021, during the crisis)**	95.1	0-6m: 59.1	At birth: 25.8<br>At <1m: 20.1<br>At <6m: 13.6<br>6-12m: 7.3<br>At >12m: 7.1	>12m: 19	At birth: 29	At 4m: 14.8<br>4-6m: 37<br>At 6m: 47.1	8.4	0.5	6.7	16.8	8.9	[4]
**Lebanon (2021, Unicef report)**	NA	NA	NA	47	NA	NA	7	NA	NA	NA	NA	[66]
**Lebanon (2021)**	84.6	0-6m: 32.4	NA	12-23m: 21.9	<6m: 39	6-8m: 78.5	7	3.7	1.8	3.7	1.2	[30]
**Lebanon (2019, before the crisis)**	NA	At 40d: 27<br>At 6m: 30	Birth-40d: 52.9<br>40d-6m: 21.9<br>At 4m: 13.8<br>At >6m: 10.7	>6m: 23	At birth: 10.6	At <4m: 1.3<br>4-6m: 39.3<br>At 6m:39.1	9.3	9.3	6.25	24.45	NA	[63]
**Lebanon (2016)**	NA	At ≤1m: 62.2<br>2-3m: 20.83<br>4-6m: 16.54	At 1d: 51.6	>6m: 0.4	NA	NA	NA	NA	NA	NA	NA	[61]
**Lebanon (2011-2012)**	89.5	At 40d: 41.5<br>At 6m: 12.3	At 40d: 20.2	NA	At 40d: 38.1<br>At 6m: 38.4	At 6m: 40.1	NA	NA	NA	NA	NA	[31]
**Lebanon (2011-2012)**	NA	NA	NA	NA	NA	NA	NA	NA	NA	6.5	2.7	[110]
**Lebanon (2010)**	NA	At <2: 40<br>4-5m: 2	NA	NA	NA	NA	NA	NA	NA	NA	NA	[62]

Countries	Prevalence of infant feeding						Prevalence of malnutrition					References
	EvBF (%)	EBF (%)	BOT (%)	CBF (%)	MixMF (%)	ISSSF (%)	Stunting (%)	Under-weight (%)	Wasting (%)	Overweight (%)	Obesity (%)	
**Lebanon (2003-2004)**	NA	NA	NA	NA	NA	At 4m: 41.6<br>At 5m: 11.3<br>At 6m: 13.4	NA	NA	NA	NA	NA	[65]
**Lebanon (2000-2004)**	NA	NA	NA	In 2000:<br>At 1y: 34.7<br>At 2y: 10.6	NA	NA	In 2004: 16.5	NA	In 2004: 6.6	In 2004: 16.7	NA	[64]
**Lebanon (UNICEF/WHO/ World Bank database, 2000-2020)**	NA	NA	NA	In 2004: 12-23m: 14.3	NA	NA	In 2000: 16<br>In 2004: 16.5<br>In 2020: 10.4	In 2004: 4.2	In 2004: 6.6	In 2000: 17.6<br>In 2004: 16.7<br>In 2020: 19.7	NA	[35,37,92,99,104, 106]
**Libya (2016-2017)**	NA	38	NA	NA	NA	NA	NA	NA	NA	NA	NA	[67]
**Libya (2014)**	NA	NA	NA	NA	NA	NA	38.1	NA	10.2	29.6	NA	[105]
**Libya (1998)**	95.6	0-5m: 44.6	At 3m: 27	6-12m: 25.1	At 3m: 26.5	NA	NA	NA	NA	NA	NA	[18]
**Libya (1995)**	NA	NA	NA	NA	NA	NA	20.7	NA	NA	NA	NA	[119]
**Libya (1995)**	NA	NA	NA	NA	NA	NA	20.7	4.3	3.7	16.2	NA	[101]
**Libya (UNICEF/WHO/ World Bank**	NA	NA	NA	NA	NA	NA	In 2020: 43.5	In 2014: 11.7	In 2014: 10.2	In 2020: 25.4	NA	[35,37,92,99,104, 106]

Countries	Prevalence of infant feeding						Prevalence of malnutrition					References
	EvBF (%)	EBF (%)	BOT (%)	CBF (%)	MixMF (%)	ISSSF (%)	Stunting (%)	Under-weight (%)	Wasting (%)	Overweight (%)	Obesity (%)	
**database, 2000-2020)**												
**Morocco (2016)**	94.7	57.23	0-6m: 30.3<br>6-12m: 17.5	NA	At birth: 39.4	NA	15.4	6.25	3.25	NA	NA	[68]
**Morocco (2003-2017)**	NA	In 2017: 35	NA	In 2017:<br>Up to 1y: 64.9<br>Up to 2y: 29.7	NA	In 2003: 84.4	In 2017: 15.1	NA	In 2017: 2.6	In 2017: 10.9	NA	[69]
**Morocco (UNICEF/WHO/ World Bank database, 2000-2020)**	NA	In 2017: 35	NA	In 2003: 35.1	NA	In 2003: 84.4	In 2020: 12.9	In 2017: 2.6	In 2017: 2.6	In 2020: 11.3	NA	[35,37,92,99,104, 106]
**Oman (2016-2017)**	NA	0-6m: 29	0-6m: 44	Up to 1y: 79<br>Up to 2y: 51	NA	NA	NA	NA	NA	NA	NA	[70]
**Oman (2016-2017)**	47	23.2	53.2	Up to 1y: 80<br>Up to 2y: 47.3	NA	95.3	11.4	11.2	9.3	4.2		[29]
**Oman (2017)**	NA	23.2	NA	Up to 1y: 80<br>Up to 2y: 47.3	NA	95.3	11.4	NA	9.3	4.2	NA	[71]

Countries	Prevalence of infant feeding						Prevalence of malnutrition					References
	EvBF (%)	EBF (%)	BOT (%)	CBF (%)	MixMF (%)	ISSSF (%)	Stunting (%)	Under-weight (%)	Wasting (%)	Overweight (%)	Obesity (%)	
**Oman (UNICEF/WHO/ World Bank database, 2000-2020).**	NA	In 2017: 23.2	NA	NA	NA	In 2017: 95.3	In 2020: 12.2	In 2017: 11.2	In 2017: 9.3	In 2020: 4.8	NA	[35,37,92,99,104, 106]
**Pakistan (2018)**	NA	48.4	NA	Up to 1y: 68.4<br>Up to 2y: 56.5	NA	35.9	40.2	28.9	17.7	9.5	NA	[74]
**Pakistan (2018)**	NA	47.8	NA	Up to 1y: 69.6<br>Up to 2y: 53.4	NA	65.3	37.6	NA	7.1	2.5	NA	[73]
**Pakistan (2017-2018)**	NA	NA	NA	NA	NA	NA	81.1	57.3	18.2	NA	NA	[96]
**Pakistan (2014-2017)**	NA	At 3m: 74.4<br>At 6m: 30.2	NA	Up to 1y: 88.3<br>Up to 2y: 32.3	NA	At 6m: 60.4	NA	NA	NA	NA	NA	[72]
**Pakistan (2015)**	93	12	NA	NA	NA	At 4m: 21<br>At 4-6m: 55<br>>6m: 18	NA	NA	NA	NA	NA	[24]
**Pakistan (2014)**	NA	NA	NA	NA	NA	NA	38.5	33.2	19.1	NA	NA	[97]
**Pakistan (2014)**	99.9	<6m: 37	12.3	Up to 1y: 82.5<br>Up to 2y: 74.7	NA	6-8m: 70	NA	NA	NA	NA	NA	[23]

Countries	Prevalence of infant feeding						Prevalence of malnutrition					References
	EvBF (%)	EBF (%)	BOT (%)	CBF (%)	MixMF (%)	ISSSF (%)	Stunting (%)	Under-weight (%)	Wasting (%)	Overweight (%)	Obesity (%)	
**Pakistan (2006-2007)**	<24m: 97.6<br><12m: 97.8<br>12-24m: 97.4	<6m: 37.1<br>0-1m: 54.6<br>2-3m: 35.7<br>4-5m: 23.1<br>0-3m: 44.1	<24m: 32.1<br><6m: 26.8<br>6-11m: 36.8<br>12-23m: 33.2	NA	NA	NA	NA	NA	NA	NA	NA	[25]
**Pakistan (UNICEF/WHO/ World Bank database, 2000-2020)**	NA	In 2018: 47.8	NA	In 2018: 62.8	NA	In 2018: 65.3	In 2020: 36.7	In 2018: 23.1	In 2018: 7.1	In 2020: 3.4	NA	[35,37,92,99,104, 106]
**Palestine (2014-2020)**	NA	In 2020: 38.9	NA	In 2014:<br>Up to 1y: 52.9<br>Up to 2y: 11.5	NA	In 2020: 89.9	8.7	NA	In 2020: 1.3	In 2020: 8.6	NA	[75]
**Palestine (2014)**	NA	NA	NA	NA	NA	NA	NA	1.4	NA	7.3	1.5	[109]
**Palestine (2014)**	NA	NA	NA	NA	NA	NA	At 6m: 9<br>At 24m: 20	At 6m: 5<br>At 24m: 4	At 6m: 10<br>At 24m: 3	NA	NA	[109]
**Palestine (2012)**	NA	NA	NA	NA	NA	NA	19.6	NA	NA	NA	NA	[121]
**Palestine (2007)**	NA	69.7	14.3	NA	NA	NA	NA	NA	NA	NA	NA	[76]
**Palestine (2009)**	NA	44.6	NA	NA	NA	NA	15	6.1	3.5	NA	NA	[77]

Countries	Prevalence of infant feeding						Prevalence of malnutrition					References
	EvBF (%)	EBF (%)	BOT (%)	CBF (%)	MixMF (%)	ISSSF (%)	Stunting (%)	Under-weight (%)	Wasting (%)	Overweight (%)	Obesity (%)	
**Palestine (2003)**	NA	27	32	NA	41	NA	31.4	31.45	34.3	NA	NA	[78]
**Palestine (UNICEF/WHO/ World Bank database, 2000-2020)**	NA	In 2020: 38.9	NA	In 2020: 28.5	NA	In 2020: 89.9	In 2020: 7.8	NA	In 2020: 1.3	In 2020: 8.5	NA	[35,37,92,99,104, 106]
**Qatar (2017)**	94	1-3m: 40<br>4-6m: 20	NA	≥12m: 34.8	NA	NA	NA	NA	NA	NA	NA	[19]
**Qatar (2012)**	96.2	24.3	NA	Up to 1y: 4.8	25.6	NA	NA	NA	NA	NA	NA	[20]
**Qatar (2009)**	97.9	0-6m: 18.9	NA	Up to 1y: 49.9<br>Up to 2y: 45.4	NA	NA	NA	NA	NA	NA	NA	[21]
**Qatar (2012)**	NA	29.3	NA	Up to 1y: 65<br>Up to 2y: 31.9	NA	74	NA	NA	NA	NA	NA	[79]
**Qatar (UNICEF/WHO/ World Bank database, 2000-2020)**	NA	In 2012: 29.3	NA	In 2012: 46.7	NA	In 2012: 74	In 2020: 4.6	In 1995: 4.8	In 1995: 2.1	In 2020: 13.9	NA	[35,37,92,99,104, 106]
**Saudi Arabia (2019)**	NA	0-6m: 28	NA	NA	NA	NA	NA	NA	NA	NA	NA	[81]

Countries	Prevalence of infant feeding						Prevalence of malnutrition					References
	EvBF (%)	EBF (%)	BOT (%)	CBF (%)	MixMF (%)	ISSSF (%)	Stunting (%)	Under-weight (%)	Wasting (%)	Overweight (%)	Obesity (%)	
**Saudi Arabia (2018)**	NA	0-6m: 27.6	NA	Up to 2y: 20.4	74.3	NA	NA	NA	NA	NA	NA	[82]
**Saudi Arabia (2005)**	NA	NA	NA	NA	NA	NA	10.9	6.9	9.8	NA	NA	[122]
**Saudi Arabia (2004-2005)**	91.6	At birth: 88.6<br>At 1m: 49<br>At 2m: 36.1<br>At 4m: 20.5<br>At 6m: 10.2	Ever BOT:8.4<br>At birth: 11.4<br>At 1m: 51<br>At 2m: 63.9<br>At 4m: 79.5<br>At 6m: 89.9<br>At 12m: 98.2	Up to 1y: 1.8	At birth: 88.6<br>At 1m: 49<br>At 2m:36.1<br>At 4m:20.5<br>At 6m:10.2<br>At 12m:1.8<br>At 18m:0.4<br>At 24m:0.2	<4m: 4.2<br>4-6m: 81.5<br>7-12m: 14.3	NA	NA	NA	NA	NA	[80]
**Saudi Arabia (2004)**	NA	NA	NA	NA	NA	NA	9.3	NA	11.8	6.1	NA	[123]
**Saudi Arabia (UNICEF/WHO/ World Bank database, 2000-2020)**	NA	NA	NA	NA	NA	NA	In 2020: 3.9	In 2004: 5.3	In 2004: 11.8	In 2020: 7.6	NA	[35,92,99,104, 106]
**Somalia (2009-2018)**	NA	In 2018: 33.7	NA	In 2009:<br>Upt to 1y: 60.8<br>Up to 2y: 26.8	NA	In 2018: 41.2	In 2009: 25.3	NA	In 2009: 14.3	In 2009: 3	NA	[84]
**Somalia (2007-2010)**	NA	NA	NA	NA	NA	NA	31	NA	21	NA	NA	[94]

Countries	Prevalence of infant feeding						Prevalence of malnutrition					References
	EvBF (%)	EBF (%)	BOT (%)	CBF (%)	MixMF (%)	ISSSF (%)	Stunting (%)	Under-weight (%)	Wasting (%)	Overweight (%)	Obesity (%)	
**Somalia (2006)**	NA	9	NA	NA	NA	NA	42	36	13	NA	NA	[83]
**Somalia (UNICEF/WHO/ World Bank database, 2000-2020)**	NA	In 2018: 33.7	NA	In 2018: 44.6	NA	In 2018: 41.2	In 2020: 27.4	In 2009: 22.5	In 2006: 13.3	In 2020: 2.9	NA	[35,37,92,99,104, 106]
**Sudan (2018-2019)**	NA	62.31	NA	Up to 2y: 73.29	NA	NA	36.35	29.16	13.6	2.14	0.85	[85]
**South Sudan (2018)**	NA	NA	NA	NA	NA	NA	23.8	4.8	2.3	NA	NA	[95]
**Sudan (2014)**	96	NA	NA	NA	NA	NA	NA	NA	NA	NA	NA	[22]
**North Sudan (2014)**	NA	62.1	NA	Up to 2y: 64	NA	NA	42.5	32.7	21	NA	NA	[86]
**Sudan (2014)**	NA	54.6	NA	Up to 1y: 89.4<br>Up to 2y: 48.8	NA	61.2	38.2	NA	16.3	3	NA	[87]
**South Sudan (UNICEF/WHO/ World Bank database, 2000-2020)**	NA	In 2010: 44.5	NA	In 2010: 61.9	NA	In 2010: 41.6	In 2020: 30.6	In 2010: 27.7	In 2010: 22.7	In 2020: 5.7	NA	[35,37,92,99,104, 106]

Countries	Prevalence of infant feeding						Prevalence of malnutrition					References
	EvBF (%)	EBF (%)	BOT (%)	CBF (%)	MixMF (%)	ISSSF (%)	Stunting (%)	Under-weight (%)	Wasting (%)	Overweight (%)	Obesity (%)	
**Sudan (UNICEF/WHO/ World Bank database, 2000-2020)**	NA	In 2014: 54.6	NA	In 2014: 72.5	NA	In 2014: 61.2	In 2020: 33.7	In 2014: 33	In 2014: 16.3	In 2020: 2.7	NA	[35,37,92,99,104, 106]
**Syrian Arab Republic (UNICEF/WHO/ World Bank database, 2000-2020)**	NA	In 2019: 28.5	NA	In 2006: 44.7	NA	In 2019: 74.6	In 2020: 29.6	NA	In 2010: 11.5	In 2020: 18.2	NA	[35,37,92,99,104, 106]
**Syrian Arab Republic (2009-2019)**	NA	In 2019: 28.5	NA	In 2009:<br>At 1y: 55.8<br>At 2y: 24.9	NA	In 2019: 74.6	In 2010: 27.9	In 2010: 10.4	In 2010: 11.5	In 2010: 17.9	NA	[88]
**Tunisia (2018)**	NA	13.5	NA	At 1y: 45.4<br>At 2y: 18.2	NA	96.8	8.4	NA	2.1	17.2	NA	[89]
**Tunisia (UNICEF/WHO/ World Bank database, 2000-2020)**	NA	In 2018: 13.5	NA	In 2018: 12-23m: 30.3	NA	In 2018: 12-23m: 96.8	In 2020: 8.6	In 2018: 1.6	In 2018: 2.1	In 2020: 16.5	NA	[35,37,92,99,104, 106]
**United Arab Emirates (2020)**	95	0-6m: 37<br>0-3.9 m:45<br>4-5.9 m: 26<br>6-11.9 m: 4	NA	>1y: 1	0-3.9m: 45<br>4-5.9 m: 48<br>6-8.9 m: 63<br>9-11.9m: 59<br>>1y: 34	<4m: 7<br><6m: 19<br>At 6m: 98	15	NA	8	7		[16]
**United Arab Emirates (2019-2020)**	NA	NA	NA	NA	NA	NA	10	NA	6	5	3	[124]

Countries	Prevalence of infant feeding						Prevalence of malnutrition					References
	EvBF (%)	EBF (%)	BOT (%)	CBF (%)	MixMF (%)	ISSSF (%)	Stunting (%)	Under-weight (%)	Wasting (%)	Overweight (%)	Obesity (%)	
**United Arab Emirates (2016)**	95.6	0-6m: 44.3	NA	NA	NA	≥6m: 89	NA	NA	NA	NA	NA	[17]
**Abu Dhabi, United Arab Emirates (2014-2015)**	NA	0-6m: 16.9	NA	NA	NA	NA	NA	NA	NA	NA	NA	[90]
**Yemen (2013)**	NA	NA	NA	NA	NA	NA	47	39	16	NA	NA	[93]
**Yemen (2013)**	NA	9.7	NA	At 1y: 71.2<br>At 2y: 45.3	NA	69.2	46.4	NA	16.4	2.5	NA	[91]
**Yemen (UNICEF/WHO/ World Bank database, 2000-2020)**	NA	In 2013:<br>0-5m: 9.7	NA	In 2013:<br>12-24m: 62.8	NA	In 2013:<br>6-8m: 69.2	In 2013: 46.4<br>In 2020: 37.2	NA	In 2013 16.4	In 2020: 2.7	NA	[35,37,92,99,104, 106]
**EMR (2018)**	NA	29.3	NA	NA	NA	NA	28	18	8.69	8.42		[3]
**Global (UNICEF/WHO/ World Bank database, 2000-2020**	NA	2014-2020: 44	NA	2014-2020: 65	NA	2014-2020: 73	In 2020: 22	In 2020: 12.6	In 2020: 6.7	In 2020: 5.7	NA	[35,37,92,99,104, 106]
**East Asia and Pacific (2014-2020)**	NA	2014-2020: 31	NA	2014-2020: 58 (excluding China)	NA	2014-2020: 84	13.4	5.2	3.7	7.8	NA	[35,37,92,99,104, 106]

Countries	Prevalence of infant feeding						Prevalence of malnutrition					References
	EvBF (%)	EBF (%)	BOT (%)	CBF (%)	MixMF (%)	ISSSF (%)	Stunting (%)	Under-weight (%)	Wasting (%)	Overweight (%)	Obesity (%)	
**Latin America and Carribean (2014-2020)**	NA	2014-2020: 37	NA	2014-2020: 45	NA	2014-2020: 87	11.3	2.7	1.3	7.5	NA	[35,37,92,99,104, 106]
**Latin America and Caribbean (2015)**	NA	NA	NA	NA	NA	NA	NA	NA	NA	NA	9.2	[125]
**North America (2014-2020)**	NA	2014-2020: 26	NA	2014-2020: 12	NA	NA	3.2	0.7	0.2	9.1	NA	[35,37,92,99,104, 106]
**South Asia (2014-2020)**	NA	2014-2020: 57	NA	2014-2020: 78	NA	2014-2020: 58	31.8	27.4	14.7	2.2	NA	[35,37,92,99,104, 106]
**Central Europe and the Baltics (2020)**	NA	NA	NA	NA	NA	NA	4.5	NA	NA	6.6	NA	[35,37,92,99,104, 106]
**Europe and Central Asia (2014-2020)**	NA	2014-2020: 41	NA	2014-2020: 50	NA	2014-2020: 76	5.7	NA	NA	7.9	NA	[35,37,92,99,104, 106]
**Central and Eastern Europe and Central Asia (2015)**	NA	NA	NA	NA	NA	NA	NA	NA	NA	NA	10.9	[125]
